# Physiological Responses and Transcriptomic Characteristics of Exogenous Trehalose Alleviating Cadmium Toxicity in Maize Seedlings

**DOI:** 10.3390/plants15203117

**Published:** 2026-10-12

**Authors:** Cong Li, Jintao Zhao, Xinxin Dong, Shuang Yang, Yijun Tang, Wenjia Huang, Jingwei Chen, Qingzhuang Qiao, Jialun Zhu, Yanye Ruan, Xiaomei Dong, Jinjuan Fan

**Affiliations:** 1 College of Bioscience and Biotechnology, Shenyang Agricultural University, Shenyang 110866, China; 2 Shenyang City Key Laboratory of Maize Genomic Selection Breeding, Shenyang 110866, China; 3 Shenyang Rural Revitalization and Development Center, Shenyang 110034, China; 4 Department of Resources and Environment, Zunyi Normal College, Zunyi 563006, China

**Keywords:** cadmium, trehalose, maize, physiological responses, transcriptome

## Abstract

Cadmium (Cd) contamination poses a severe ecological risk to ecosystems. Trehalose (TR) is a non-reducing disaccharide which can mitigate the adverse effects of heavy metals. The physiological responses and transcriptomic characteristics underlying TR-mediated heavy metal tolerance remain unclear in maize. In this study, maize seedlings were exposed to 200 mg L^−1^ CdCl_2_·2.5H_2_O with or without 23 mM exogenous TR for 5 days. By phenotypic observation, although TR supplementation could not fully restore maize growth under Cd stress, it significantly alleviated Cd-induced inhibition, increasing shoot and root dry weight by 33.80% and 36.67%, respectively, compared with Cd treatment alone. Exogenous TR also improved the photosynthesis under Cd stress. Cd stress triggered excessive ROS accumulation and membrane lipid peroxidation, whereas trehalose application alleviated oxidative damage by significantly reducing $O_{2}^{-}$, H_2_O_2_, and MDA contents while further enhancing SOD and CAT activities. Compared with Cd treatment, cell-wall fractionation revealed a 20.53% increase in root cellulose under Cd+TR combined treatment. Further, exogenous TR reduced Cd concentrations in roots and shoots by 19% and 33%, respectively. RNA-seq identified enriched GO categories related to cell communication, signal transduction, amino acid transmembrane transport, and membrane components, suggesting possible involvement of these processes in the TR response. These findings collectively demonstrate that exogenous trehalose enhances maize cadmium tolerance through coordinated physiological responses and transcriptional adjustments.

## 1. Introduction

Soil heavy metal pollution, largely caused by human activities, including sewage irrigation and irrational chemical fertilizer application, has become a serious global ecological threat [1]. Cadmium (Cd) has attracted particular attention because of its high toxicity, strong mobility, and persistence in the environment. After entering the soil, Cd is readily absorbed by plant roots and accumulates in plant tissues, thereby inhibiting crop growth and development and posing risks to human health through the food chain [2]. Cropland acidification accelerates Cd accumulation in edible rice and increases the long-term food-safety risk of paddy production [3]. Consequently, reducing Cd accumulation in crops and enhancing plant tolerance to Cd stress have become key research priorities in plant stress physiology and agricultural environmental safety.

Cd stress progressively reduces the aboveground and belowground biomass of maize seedlings and causes visible root blackening as exposure increases [4]. Root morphological traits are closely correlated with Cd accumulation and the bioconcentration factor in maize genotypes [5]. Meanwhile, Cd also causes typical toxicity symptoms such as leaf chlorosis, plant dwarfing, and biomass reduction. Physiologically and biochemically, cadmium suppresses chlorophyll formation, impairs chloroplast structure, and reduces PSII activity and photosynthetic capacity. Cd accumulation in chloroplasts alters photosynthetic electron transport and changes the donor- and acceptor-side limitations of PSI and PSII in young maize plants [6]. Cd exposure disrupts chlorophyll synthesis, photosynthetic ultrastructure, and photosystem function in maize seedlings [7]. Reactive oxygen species (ROS) are regarded to be the main mediator of the oxidative stress generated by cadmium exposure [8], exacerbating membrane lipid peroxidation and breaking cell membrane integrity and redox balance [9]. Plants deploy diverse strategies to mitigate cadmium toxicity. To lessen the detrimental consequences of ROS, plants in harsh conditions evolve a system that involves increased levels of non-enzymatic antioxidants, including ascorbate (AsA) and glutathione (GSH) [10], and enhanced activities of important antioxidant enzymes, such as catalase, peroxidase and superoxide dismutase [11], as well as enzymes participating in the AsA–GSH cycle [12]. Meanwhile, plants can limit cadmium absorption and transport, increase cell-wall retention, activate antioxidant systems, accumulate osmoregulatory substances, and sequester cadmium in vacuoles. External metal exclusion and internal detoxification involve cell-wall modification, activation of ROS-scavenging systems, organic-acid secretion, and sequestration of toxic ions in vacuoles [13]. *ZmMTP1-1* and *ZmMTP1-2* contribute to Cd tolerance through coordinated metal transport, increased antioxidant enzyme activities, and reduced oxidative damage [14]. However, such defensive responses vary greatly across plant species, varieties, and growth phases.

In recent years, the application of exogenous regulatory substances has become a widely adopted strategy for improving plant tolerance to abiotic stresses because of its simple operation and rapid physiological effect. Trehalose (TR), a non-reducing disaccharide, functions as both an osmoprotectant and a signaling molecule in plant responses to drought, salinity, cold and heavy-metal stresses [15]. Glucose-6-phosphate and UDP-glucose are converted into trehalose-6-phosphate (T6P) by the enzyme trehalose-6-phosphate synthase (TPS), and then trehalose-6-phosphate phosphatase (TPP) dephosphorylates T6P to generate TR in plants [15]. Trehalose exerts protective effects via stabilizing cell membranes and protein conformation, sustaining osmotic equilibrium, and triggering antioxidant responses, as well as modulating sugar metabolism and stress defense pathways. Trehalose regulates osmotic pressure, stabilizes proteins, and participates in carbon-allocation signaling during abiotic stress [16]. Increased trehalose biosynthesis through *AmTPS6* limits Cd influx and protects photosynthetic structures in *Avicennia marina* under Cd stress [17]. For example, exogenous trehalose reduced NaCl-induced growth inhibition in rice and enhanced antioxidant-related protection [18,19]. In maize seedlings, trehalose application alleviated salt-induced membrane lipid peroxidation by reducing MDA accumulation [20]. In quinoa plants under water deficit, trehalose enhanced antioxidant defense and osmolyte accumulation [21]. Under heavy-metal stress, trehalose enhanced copper tolerance in rice by strengthening antioxidant defense and reducing oxidative damage [22]. More directly, trehalose can mitigate Cd toxicity by reducing Cd mobility and maintaining redox homeostasis in rice seedlings [1]. However, comparable evidence in maize under Cd stress remains limited, and the genes triggered by TR in maize remain insufficiently characterized.

Maize, as a critical source of food, animal feed, and industrial raw materials, is a major crop in the world. Ensuring stable and high yield of maize is of great significance for national food security. In the study, we applied exogenous trehalose under Cd stress to systematically analyze its effects on maize seedling growth and morphology, photosynthetic characteristics, antioxidant systems, osmotic adjustment, Cd uptake and translocation, root cell wall components, and endogenous trehalose metabolism. In combination with transcriptome sequencing, key genes and regulatory pathways associated with trehalose-mediated alleviation of Cd toxicity were identified and analyzed. This study provides evidence for TR-mediated alleviation of Cd injury in maize seedlings.

## 2. Results

### 2.1. TR Partially Restores Seedling Growth Under Cd Stress

**Figure 1:**
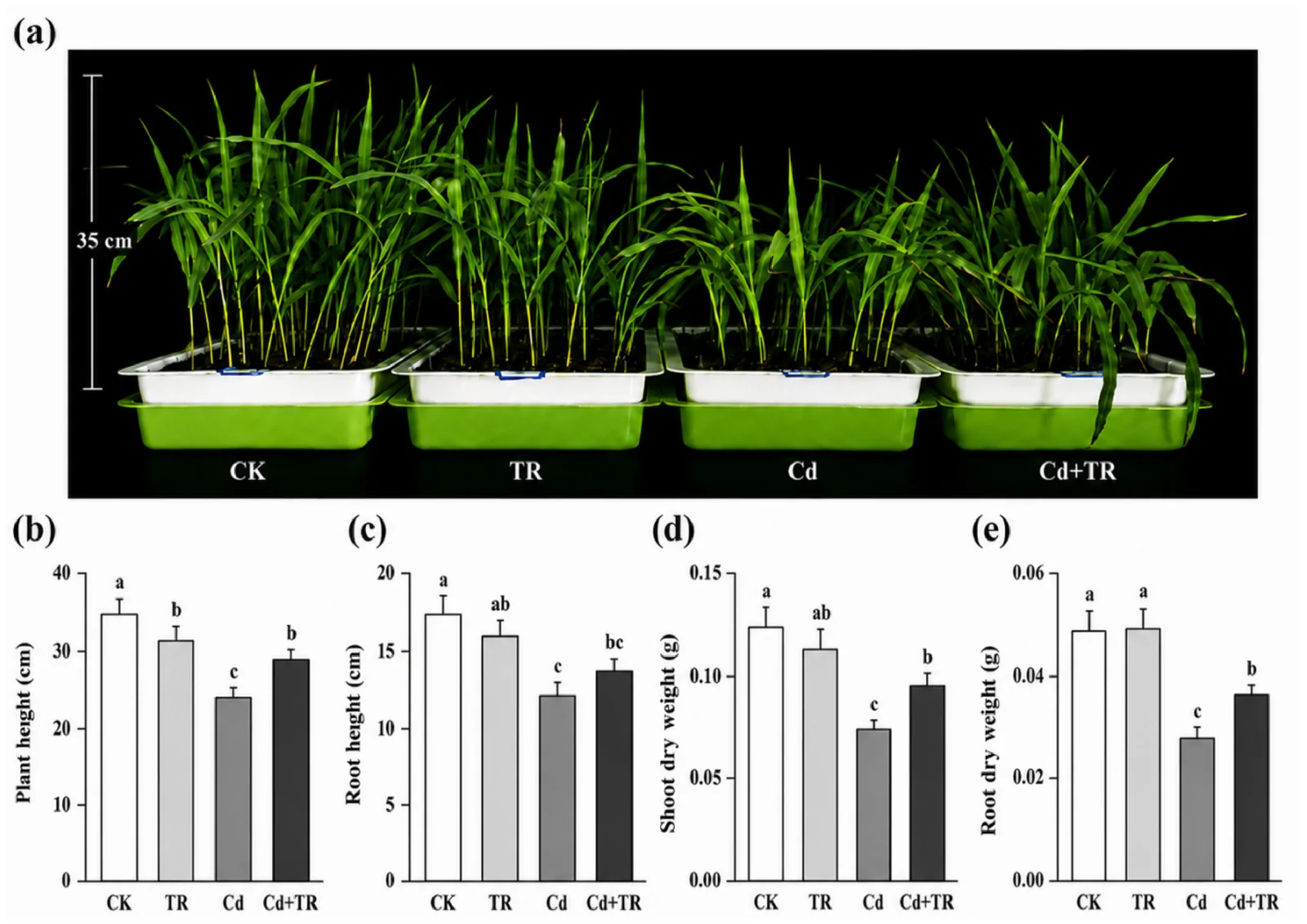
Effects of exogenous trehalose on the growth of maize seedlings under Cd stress. (**a**) Phenotypes of maize seedlings under CK, TR, Cd, and Cd+TR treatments; (**b**) plant height; (**c**) root length; (**d**) shoot dry weight; (**e**) root dry weight. CK, control; TR, 23 mM trehalose; Cd, 200 mg L^−1^ CdCl_2_·2.5H_2_O; Cd+TR, 200 mg L^−1^ CdCl_2_·2.5H_2_O + 23 mM trehalose. Values are means ± SE (*n* = 3). Different lowercase letters indicate significant differences according to Duncan’s multiple range test (*p* < 0.05).

The effects of trehalose on the growth of maize seedlings under Cd stress through phenotypic observation were investigated. As shown in Figure 1a, significant differences were observed among treatments after 5 days of trehalose (TR) treatment, Cd treatment, and combined Cd+TR treatment. Under Cd stress, seedling growth and development were severely inhibited, characterized by stunted growth, slender stems, yellowing and wilting leaves, and dried, curled leaf tips. However, under Cd+TR, seedlings showed thicker stems and lighter leaf yellowing compared to those subjected to Cd treatment alone. Then, the plant height, root length, shoot dry weight, and root dry weight of maize seedlings under control, TR treatment, Cd treatment, and Cd+TR treatment were investigated. The plant heights of maize seedlings under control, all (TR, Cd, and Cd+TR) treatments were 33.33 cm, 30.83 cm, 21.93 cm, and 29.90 cm, respectively (Figure 1b). Compared with the control, all (TR, Cd, and Cd+TR) treatments significantly reduced seedling height by 7.50%, 34.20%, and 10.29%, respectively. As shown in Figure 1c, under Cd+TR treatment, root length was significantly lower than the control, but showed a slight increase compared to Cd treatment. Cd treatment significantly reduced shoot dry weight by 38.79%. The Cd+TR treatment also resulted in significantly lower shoot dry weight than the control, but showed a significant increase of 33.80% compared to Cd treatment alone (Figure 1d). Under Cd treatment, root dry weight was reduced by 38.78% compared to the control. The Cd+TR treatment also resulted in significantly lower root dry weight than the control, but increased significantly by 36.67% compared to Cd treatment alone (Figure 1e).

### 2.2. TR Modulates Photosynthetic Capacity Under Cd Stress

**Figure 2:**
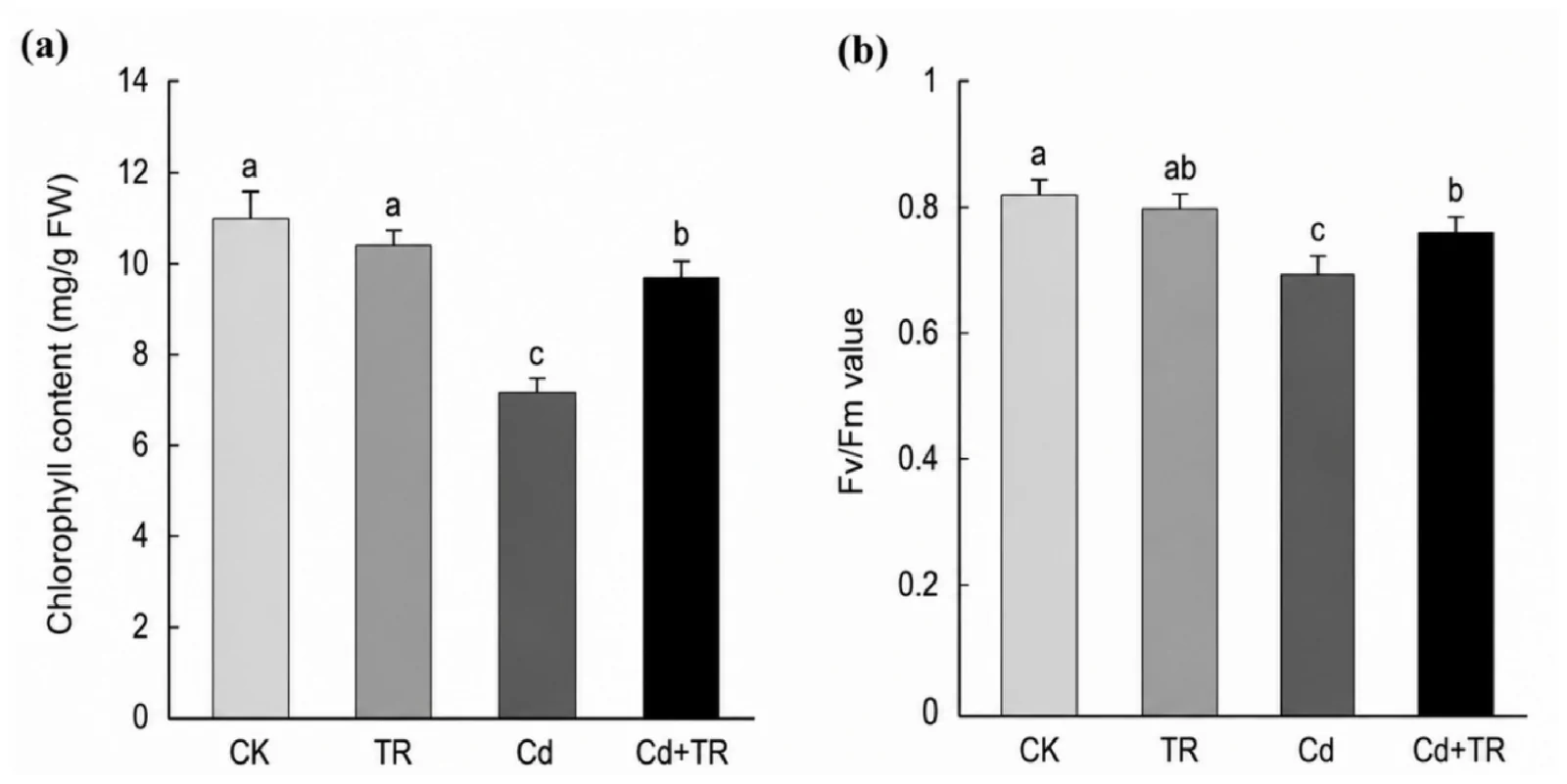
Effects of exogenous trehalose on photosynthetic characteristics of maize seedlings under Cd stress. (**a**) Chlorophyll content. (**b**) Maximum photochemical efficiency of PSII (Fv/Fm). CK, control; TR, 23 mM trehalose; Cd, 200 mg L^−1^ CdCl_2_·2.5H_2_O; Cd+TR, 200 mg L^−1^ CdCl_2_·2.5H_2_O supplemented with 23 mM trehalose. Values are presented as means ± SE (*n* = 3). Different lowercase letters above the bars indicate significant differences among treatments according to Duncan’s multiple range test (*p* < 0.05).

Chlorophyll content is directly related to the photosynthetic assimilation in plants. As shown in Figure 2a, chlorophyll content in maize seedling leaves under Cd treatment was significantly reduced by 32.63%, showing significant differences compared with the other three treatments. Under trehalose treatment, chlorophyll content decreased slightly, with no significant difference compared to the control. Under combined Cd+TR treatment, leaf chlorophyll content was significantly higher than that under Cd treatment alone, increasing by 32.76%, but still remained lower than both the control and TR treatment. Under Cd treatment, Fv/Fm decreased significantly to 0.71, showing significant differences compared with the control, TR treatment, and Cd+TR treatment (Figure 2b).

### 2.3. TR Elevates Endogenous Trehalose and Trehalase Activity and Curtails Cd Accumulation

**Figure 3:**
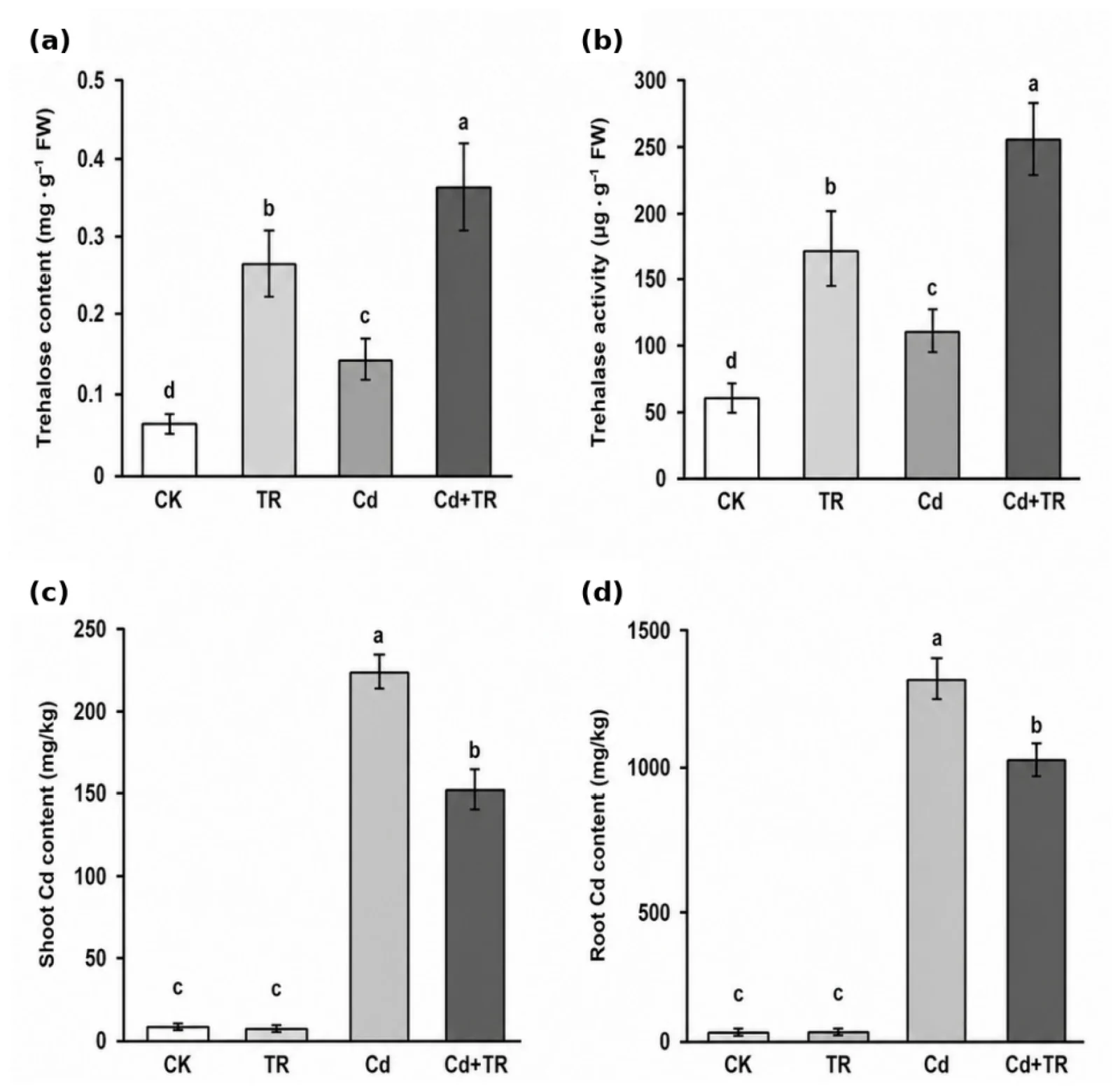
Effects of exogenous trehalose on Cd content, trehalose content and trehalase activity in maize seedlings under Cd stress. (**a**) trehalose content in maize seedlings; (**b**) trehalase activity in maize seedlings; (**c**) Cd content in maize seedlings; (**d**) Cd content in maize roots; CK, control; TR, 23 mM trehalose; Cd, 200 mg L^−1^ CdCl_2_·2.5H_2_O; Cd+TR, 200 mg L^−1^ CdCl_2_·2.5H_2_O + 23 mM trehalose. Values are means ± SE (*n* = 3). Different lowercase letters indicate significant differences according to Duncan’s multiple range test (*p* < 0.05).

Compared with the control group, the trehalose content in the leaves of maize seedlings under Cd treatment, trehalose treatment, and Cd+TR combined treatment was significantly increased by 121.13%, 295.36%, and 448.45%, respectively; among them, the increase range of trehalose treatment was higher than that of Cd treatment, with a significant difference between the two. The trehalose content under the combined treatment was the highest, which was 448.45%, 38.72%, and 148.02% higher than that of the control group, trehalose treatment, and Cd treatment, respectively (Figure 3a). Compared with the control group, the trehalase activity in the leaves under Cd treatment, trehalose treatment, and Cd+TR combined treatment was significantly increased by 75.92%, 176.90%, and 319.44%, respectively; the trehalase activity under the combined treatment was the highest, which was 319.44%, 51.48%, and 138.44% higher than that of the control group, trehalose treatment, and Cd treatment, respectively (Figure 3b). As shown in Figure 3c and Figure 3d, compared with the control, Cd treatment and Cd+TR combined treatment significantly increased the Cd content, but the combined treatment also significantly decreased the Cd content by 33.25% and 18.92% in shoots and roots, respectively, compared with Cd treatment. The Cd transfer coefficient was 0.09, 0.09, 0.17, and 0.14 under the control and treatments, respectively (Supplementary Figure S1). Cd treatment significantly increased the transfer coefficient compared with the control, while trehalose treatment had no significant difference with the control, and the combined treatment significantly reduced it compared with Cd treatment.

### 2.4. TR Mitigates Oxidative Damage of Maize Seedlings Under Cd Stress

**Figure 4:**
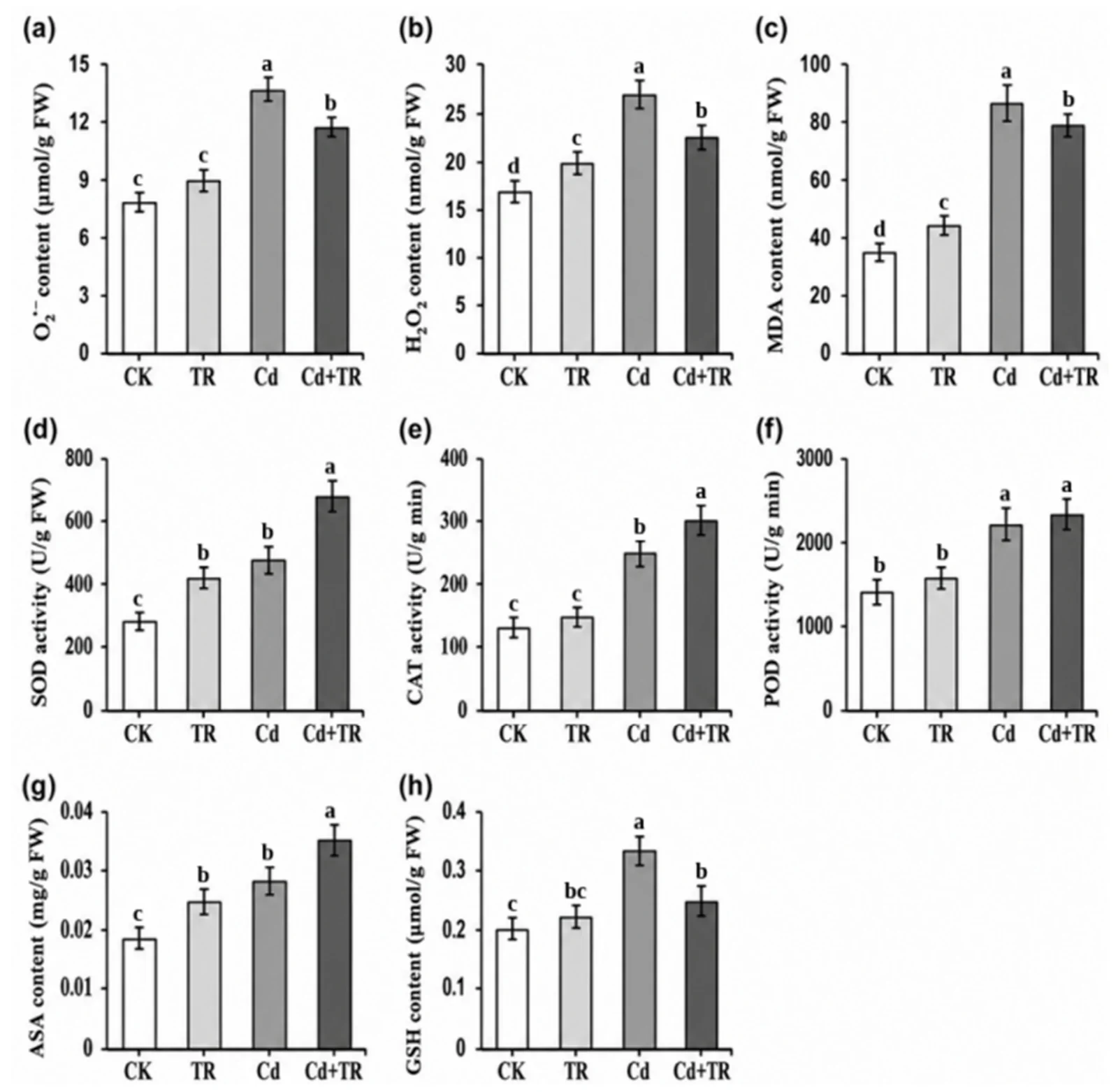
Effects of exogenous trehalose on oxidative damage and antioxidant defense in maize seedlings under Cd stress. (**a**) $O_{2}^{\cdot -}$ content in maize seedlings; (**b**) hydrogen peroxide content in maize seedlings; (**c**) malondialdehyde content; (**d**) SOD activity in maize seedlings; (**e**) CAT activity in maize seedlings; (**f**) POD activity in maize shoot; (**g**) AsA content in maize shoot; (**h**) GSH content in maize shoot. CK, control; TR, 23 mM trehalose; Cd, 200 mg L^−1^ CdCl_2_·2.5H_2_O; Cd+TR, 200 mg L^−1^ CdCl_2_·2.5H_2_O + 23 mM trehalose. Values are means ± SE (*n* = 3). Different lowercase letters indicate significant differences according to Duncan’s multiple range test (*p* < 0.05).

Elevated ROS content can reflect the degree of stress-induced damage to the plant. Compared with the control, Cd treatment significantly increased the contents of O_2_•^−^ and H_2_O_2_ in maize seedling leaves by 80.03% and 72.92%, respectively, whereas trehalose (TR) treatment alone only slightly elevated H_2_O_2_. Although the contents of O_2_•^−^ and H_2_O_2_ in leaves under combined Cd+TR treatment remained significantly higher than those in the control, they were significantly decreased by 16.18% and 17.47%, respectively, compared with Cd treatment alone (Figure 4a,b). Compared with the control, MDA content in maize seedling leaves under both Cd and trehalose treatments increased significantly. Under combined Cd+TR treatment, leaf MDA content was also significantly higher than that of the control, but reduced by 8.28% compared to Cd treatment alone, reaching a significant difference level (Figure 4c). As shown in Figure 4d–f, antioxidant enzyme activity analysis showed that Cd treatment significantly induced the activities of SOD, CAT and POD, which increased by 61.79%, 93.55%, and 62.68%, respectively, compared with the control. Compared with Cd treatment alone, combined Cd+TR treatment further significantly increased SOD and CAT activities (by 47.27% and 23.33%, respectively), and also showed an increasing trend for POD activity. AsA and GSH are important non-enzymatic antioxidants involved in plant redox homeostasis. The results of non-enzymatic antioxidants AsA and GSH (Figure 4g,h) showed that Cd treatment significantly increased the contents of AsA and GSH. Compared with Cd treatment alone, combined Cd+TR treatment significantly increased AsA content by 23.81%, while significantly decreasing GSH content by 25.75%.

### 2.5. TR Modifies Root Cell Walls

**Figure 5:**
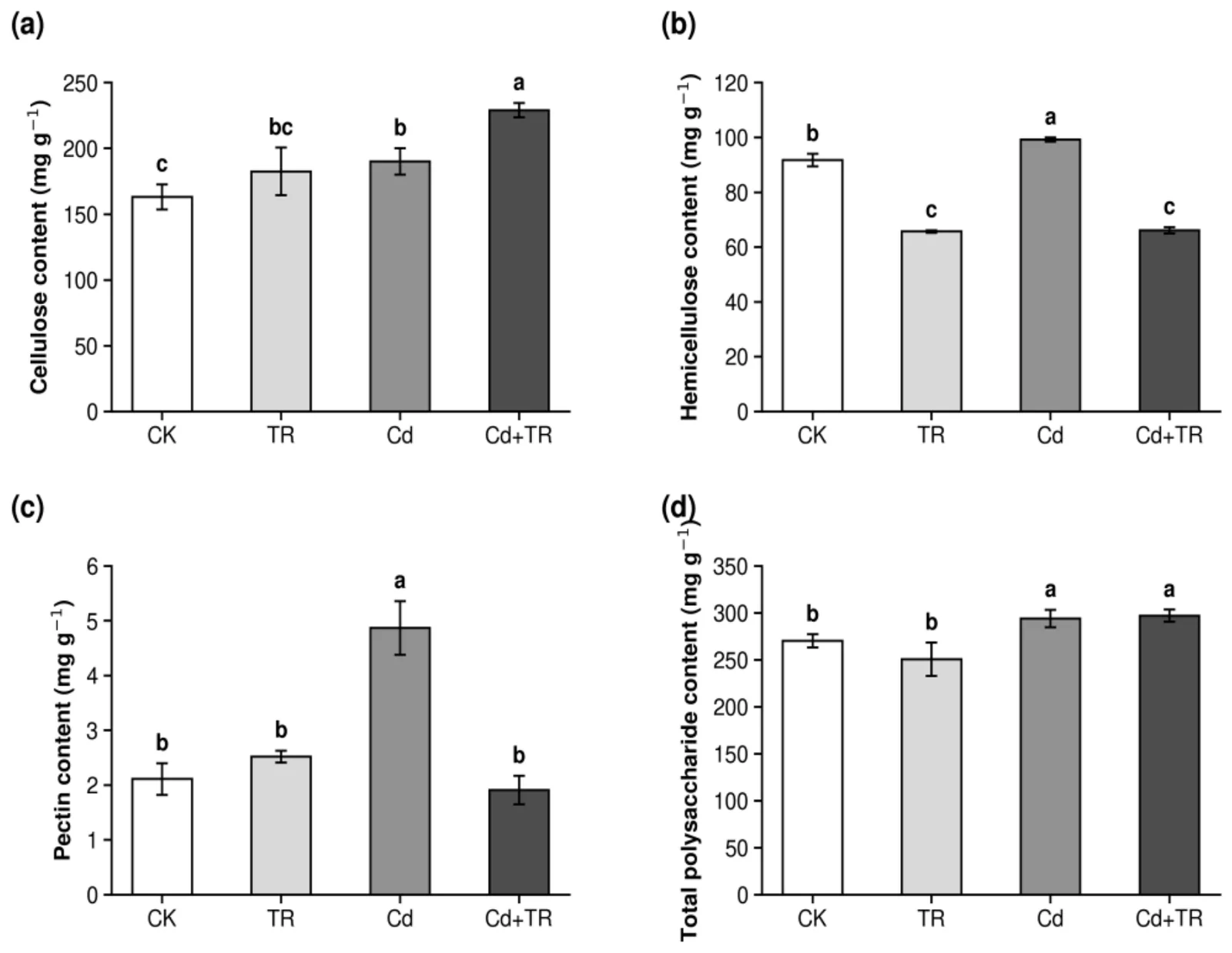
Effects of exogenous trehalose on polysaccharide components in root cell wall of maize seedlings under Cd stress. (**a**) Cellulose content; (**b**) hemicellulose content; (**c**) pectin content; (**d**) total polysaccharide content. CK, control; TR, 23 mM trehalose; Cd, 200 mg L^−1^ CdCl_2_·2.5H_2_O; Cd+TR, 200 mg L^−1^ CdCl_2_·2.5H_2_O + 23 mM trehalose. Values are means ± SE (*n* = 3). Different lowercase letters indicate significant differences according to Duncan’s multiple range test (*p* < 0.05).

As shown in Figure 5, compared with the control, cellulose, hemicellulose, and pectin contents in the maize root cell wall all increased significantly under Cd stress, and the total cell wall polysaccharide content was higher than that of the control. Under single trehalose treatment, cellulose and pectin contents were slightly higher than those of the control, with no significant differences, while hemicellulose content decreased significantly. The total cell wall polysaccharide content was lower than that of the control, but the difference was not significant. Compared with Cd treatment, Cd+TR combined treatment increased cellulose content in the cell wall by 20.53% and decreased hemicellulose and pectin contents, both reaching significant difference levels. The total cell wall polysaccharide content was slightly higher than that under Cd treatment, but the difference was not significant. The relative contents of polysaccharide components in the maize root cell wall under various treatments are shown in Supplementary Figure S2. Under different treatments, cellulose content was consistently the highest in the maize root cell wall, followed by hemicellulose, with pectin content being the lowest. Combined with Figure 5, it can be concluded that under normal conditions, exogenous trehalose did not affect the total cell wall polysaccharide content, but altered the ratio of cellulose to hemicellulose by significantly decreasing hemicellulose content. Under Cd stress, trehalose also had no significant effect on the total cell wall polysaccharide content; instead, it increased cellulose content while decreasing hemicellulose and pectin contents, thereby changing the original polysaccharide composition ratio of the cell wall.

### 2.6. Impact of Exogenous Trehalose on Osmolyte Contents in Maize Seedlings Under Cd Stress

**Figure 6:**
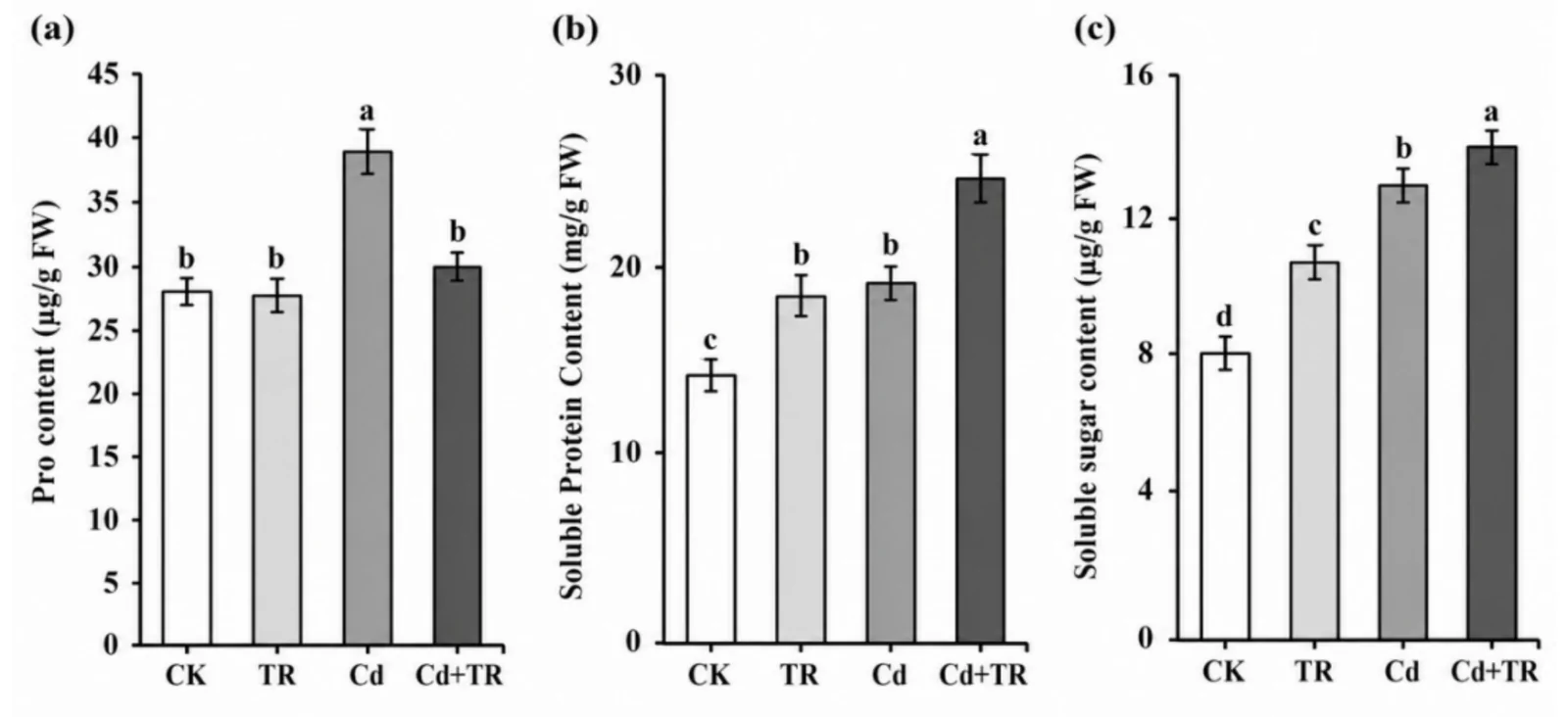
Effects of exogenous trehalose on osmotic adjustment substances in maize seedlings under Cd stress. (**a**) Proline content in maize seedlings; (**b**) soluble protein content in maize seedlings; (**c**) soluble sugar content in maize shoots. CK, control; TR, 23 mM trehalose; Cd, 200 mg L^−1^ CdCl_2_·2.5H_2_O; Cd+TR, 200 mg L^−1^ CdCl_2_·2.5H_2_O + 23 mM trehalose. Values are means ± SE (*n* = 3). Different lowercase letters indicate significant differences according to Duncan’s multiple range test (*p* < 0.05).

As important compatible solutes, proline and soluble protein accumulation is crucial for plant adaptation to abiotic stress (Figure 6a–c). Under Cd treatment, leaf proline content increased by 38.71% compared with the control, while trehalose treatment caused a slight, non-significant increase (Figure 6a). The combined Cd+TR treatment showed no significant difference from the control but significantly decreased proline content by 21.35% relative to Cd treatment alone (Figure 6a). As shown in Figure 6b, Cd treatment significantly increased leaf soluble protein content by 31.31% compared with the control. Trehalose treatment also significantly elevated soluble protein by 23.26%, with no significant difference between Cd and trehalose alone. The combined treatment further increased soluble protein by 33.38% compared with Cd treatment alone. As important osmolytes and nutrients, soluble sugar contents were significantly enhanced in maize shoots by Cd (65.57%) and trehalose (32.77%) treatments relative to the control, with a greater increase under Cd. Compared with Cd alone, the combined treatment significantly increased shoot soluble sugar content by 9.25% (Figure 6c). Thus, the TR response involved higher soluble protein and sugar contents but lower proline accumulation, rather than a uniform increase in all measured osmolytes.

### 2.7. TR Triggers Specific Transcriptional Changes

**Figure 7:**
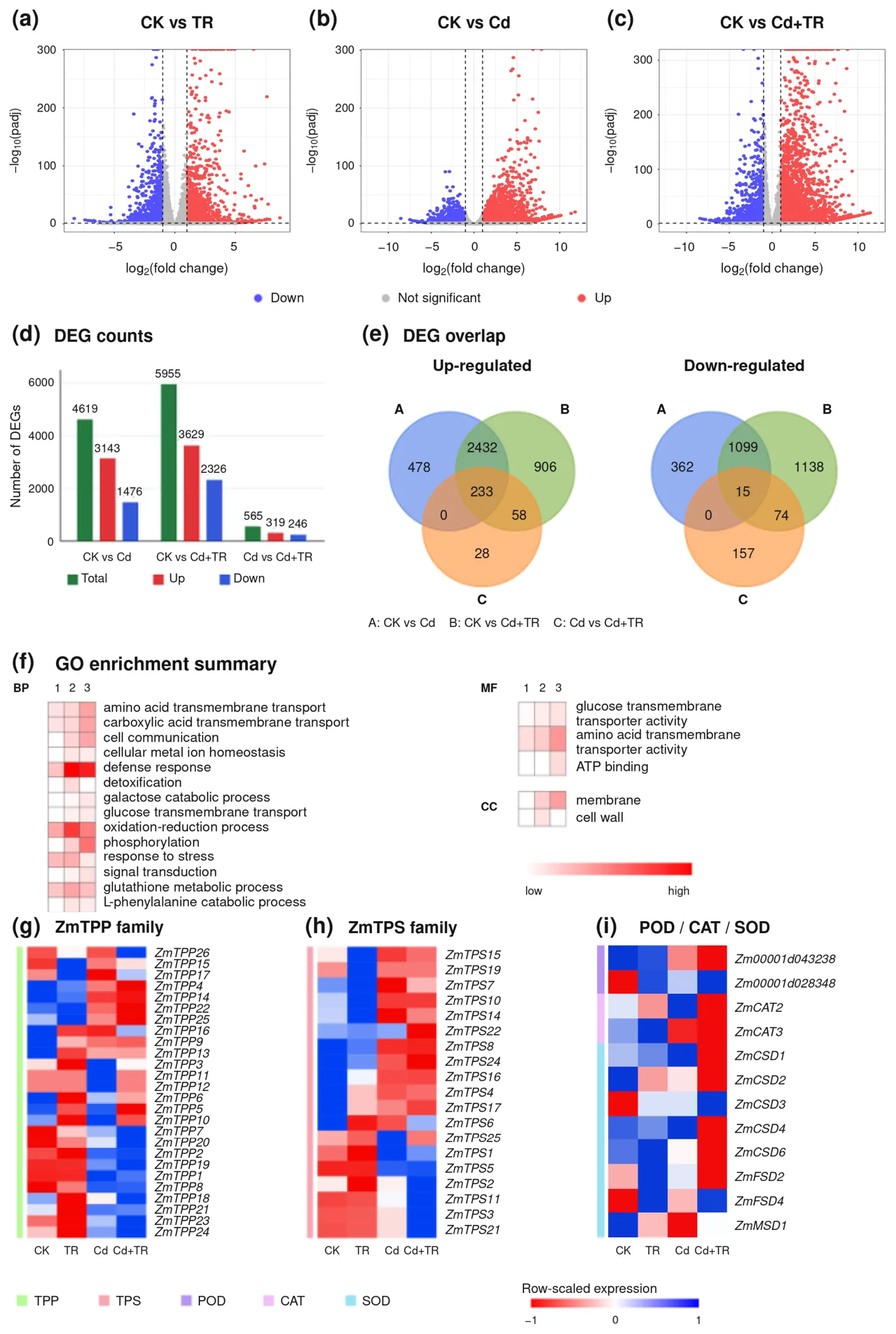
Transcriptomic responses of maize seedlings to exogenous trehalose under Cd stress. (**a**–**c**) Volcano plots of differentially expressed genes (DEGs) for (**a**) CK vs. TR, (**b**) CK vs. Cd, and (**c**) CK vs. Cd+TR. Red dots indicate significantly up-regulated genes, blue dots indicate significantly down-regulated genes, and gray dots indicate non-significant genes. In panels (**a**–**c**), the vertical dashed lines indicate log_2_(fold change) = −1 and 1, and the horizontal dashed line indicates an adjusted *p*-value of 0.05. (**d**) Total, up-regulated, and down-regulated DEG counts calculated from Supplementary Table S3. (**e**) Up- and down-regulated DEG overlaps calculated from gene identifiers in Supplementary Table S3. A, CK vs. Cd; B, CK vs. Cd+TR; C, Cd vs. Cd+TR. Numbers denote exclusive regions; circle areas are not proportional to gene counts. (**f**) GO terms reproduced from the supplied summary, with columns 1–3 retained in source order. BP, biological process; MF, molecular function; CC, cellular component. (**g**–**i**) Heatmaps of 26 *ZmTPP*, 19 *ZmTPS* and 12 POD/CAT/SOD-related genes, respectively. Columns are CK, TR, Cd, and Cd+TR, following the existing manuscript. Source row order and colors are retained: blue indicates higher and red lower row-scaled expression. CK, control; TR, trehalose; Cd, cadmium treatment; Cd+TR, combined cadmium and trehalose treatment.

To investigate the responses of gene expression associated with TR-mediated alleviation of Cd toxicity, our study performed transcriptome sequencing of twelve samples including four treatments (CK, TR, Cd, and Cd+TR) with three biological replicates per treatment (Supplementary Table S1). Differentially expressed genes (DEGs) were identified (Figure 7a–d and Supplementary Table S3). A total of 4619 DEGs were identified between CK and Cd treatments, comprising 3143 up-regulated DEGs and 1476 down-regulated DEGs. Between CK and Cd+TR treatments, a total of 5955 DEGs were identified, including 3629 up-regulated DEGs and 2326 down-regulated DEGs. Between Cd and Cd+TR treatments, a total of 565 DEGs were identified, consisting of 319 up-regulated DEGs and 246 down-regulated DEGs. Subsequently, separate Venn diagram analyses of up- and down-regulated DEGs were performed to examine the numbers of specific and common DEGs among different treatments (Figure 7e; Supplementary Table S3). Most of DEGs were commonly identified between CK_VS_Cd and CK_VS_Cd+TR. Specifically, 478 up-regulated and 362 down-regulated genes were uniquely differentially expressed in the CK_VS_Cd comparison, while 906 up-regulated and 1138 down-regulated genes were uniquely differentially expressed in the CK_VS_Cd+TR comparison.

Gene Ontology (GO) enrichment analysis was performed on differentially expressed genes (DEGs) under Cd stress and trehalose treatment (Figure 7f). For upregulated genes, the CK vs. Cd comparison showed enrichment of detoxification and defense-related GO categories, including oxidation-reduction process (GO:0055114), defense response (GO:0006952), and glutathione metabolic process (GO:0006749), all of which exhibited extremely high significance (*p* < 10^−5^) in the CK vs. Cd group, consistent with transcriptional responses related to antioxidant defense and detoxification. Trehalose treatment enhanced the enrichment of signaling- and transport-related categories, including cell communication (GO:0007154), signal transduction (GO:0007165), and amino acid transmembrane transport (GO:0003333), while membrane component (GO:0016020) terms were also significantly enriched, suggesting that these processes may participate in the TR response. For downregulated genes, the Cd-stress comparison showed enrichment of categories related to basic cellular activities, including the mitotic cell cycle (GO:0000278) and DNA-dependent DNA replication (GO:0006261). Meanwhile, pathways such as translation (GO:0006412) and ribosome biogenesis (GO:0042254) showed significant enrichment in the CK vs. TR group, indicating a potential inhibitory effect of TR on the protein synthesis system.

To connect the transcriptomic patterns with the physiological changes described above, we focused on genes involved in trehalose metabolism and antioxidant defense (Figure 7g–i). Members of the *ZmTPP* and *ZmTPS* families responded differently across treatments, and several genes showed higher transcript abundance under Cd+TR than under Cd alone. The qRT-PCR of *ZmTPP8* and *ZmTPP23* showed expression patterns consistent with the RNA-seq results (Figure S4 and Supplementary Table S2). The expression profiles of SOD-, POD-, and CAT-related genes also broadly matched the measured changes in enzyme activity and ROS levels, suggesting a transcriptional association with the antioxidant response observed after trehalose application.

## 3. Discussion

Cd exposure clearly suppressed seedling growth and photosynthetic performance, as reflected by reduced biomass accumulation, chlorophyll content, and Fv/Fm, and this inhibition was accompanied by increased ROS accumulation and lipid peroxidation. In contrast, Cd+TR partially restored biomass accumulation, chlorophyll content, and PSII efficiency, indicating that TR maintains the functional status of photosynthetic tissues under Cd stress. A comparable protective effect was recently reported in the mangrove *Avicennia marina*, where exogenous trehalose improved photosynthetic performance and stabilized chloroplast and nuclear ultrastructure under Cd exposure [17]. Taken together, these results suggest that the partial recovery of photosynthetic function in maize was associated with lower tissue Cd content and reduced oxidative damage.

Cd restriction appears to be a central component of TR-mediated tolerance. In our experiment, TR reduced Cd concentrations in both roots and shoots and lowered the Cd transfer coefficient compared with Cd treatment alone, consistent with altered Cd accumulation and partitioning. This pattern is consistent with the finding that exogenous trehalose inhibited Cd^2+^ influx in *A. marina* roots and affected Cd transport-related genes [17], and also agrees with reports that trehalose reduced Cd mobility in rice seedlings [1]. Similarly, Kaya et al. [23] showed that the beneficial effect of trehalose/NO on pepper under Cd stress was closely associated with Cd sequestration in the cell-wall fraction, thereby restricting Cd transfer to sensitive cellular compartments. The 20.53% increase in root cellulose under Cd+TR suggests that exogenous trehalose may promote cell-wall remodeling. However, increased cellulose content alone does not demonstrate greater Cd binding or retention in the cell wall; direct quantification of cell-wall-bound Cd (e.g., by cell fractionation or energy-dispersive X-ray spectroscopy) would be required to confirm this proposed mechanism.

The reduction of oxidative damage under Cd+TR treatment was accompanied by enhanced SOD and CAT activities and higher AsA accumulation, while the contents of O_2_•−, H_2_O_2_, and MDA were lower than under Cd alone. This response is consistent with improved antioxidant defense, although the measurements do not resolve ROS production or signaling dynamics. Research in melon demonstrated that trehalose-induced tolerance in seedlings involves H_2_O_2_ transport through the aquaporin CmPIP2;3 and subsequent activation of antioxidation [24]. Although aquaporin-mediated H_2_O_2_ transport was not directly examined in the present study, the enrichment of signal transduction and membrane-related categories, together with enhanced antioxidant enzyme activity, supports the possibility that TR fine-tunes ROS signaling while limiting oxidative injury under Cd stress. GSH nevertheless decreased by 25.75% relative to Cd alone, indicating that TR altered the balance among antioxidant components rather than increasing all antioxidant pools. A comparable pattern was reported in Cu-stressed rice: trehalose reduced stress-induced GSH accumulation while increasing AsA and improving redox status [22]. Changes in GSH consumption, recycling, or phytochelatin synthesis could contribute, but GSSG, phytochelatins, and the relevant metabolic fluxes were not measured.

Changes in osmolyte contents may also contribute to the protective effect of TR. Exogenous trehalose increased trehalose, proline, soluble protein, and soluble sugar levels in okra under salt and drought stress [25]. Trehalose treatment regulated soluble sugars, soluble proteins and proline in salt-stressed tomato seedlings and strengthened cellular osmotic protection [26]. Under Cd stress, the accumulation of proline and soluble sugars often reflects both adaptive osmotic regulation and stress-induced metabolic disturbance. In this study, Cd+TR reduced proline accumulation relative to Cd alone, while markedly increasing soluble sugars and soluble proteins. A reduction in stress-induced proline accumulation was also observed after trehalose pretreatment in Cu-stressed rice [22]. This shift suggests that TR partly relieved the need for excessive proline accumulation while maintaining carbon availability and protein protection. Moreover, Cd-induced soluble sugar accumulation in maize has been associated with increased sucrose hydrolysis and impaired growth [27]. The strong increase in soluble sugars is particularly relevant because trehalose metabolism is closely connected with sugar signaling and carbon allocation and may help maintain membrane hydration and protein stability under stress. These physiological changes are consistent with the involvement of carbohydrate-related processes and the expression responses of *ZmTPP* and *ZmTPS* members shown in Figure 7, indicating that exogenous TR may reinforce endogenous trehalose/carbohydrate metabolic networks in maize seedlings. Under Cd+TR, both trehalose content and trehalase activity increased. This pattern may reflect greater trehalose turnover, as concurrent increases in trehalose accumulation and the activities of synthetic and degradative enzymes have been observed in heat-stressed fungi [28]. Trehalase may also contribute to stress responses in plants. *AtTRE1* enhances ABA-induced stomatal closure and drought tolerance in *Arabidopsis* [29]. The findings provide a possible explanation for the concurrent increases observed here, but whether similar processes contribute to the response of maize to Cd remains should be explored in the future.

At the transcriptome level, the distinction between Cd and Cd+TR treatments suggested candidate processes associated with TR. Cd alone was associated with enrichment of oxidation-reduction, defense, and glutathione-related categories, reflecting a stress-response state dominated by detoxification. In contrast, Cd+TR was associated with enriched categories related to cell communication, signal transduction, amino acid transmembrane transport, and membrane components. Amino acid transport may support glutathione/phytochelatin synthesis and protein repair. Phloem-specific overexpression of *AtOPT6* altered glutathione, phytochelatin, and Cd distribution and increased both phytochelatin and Cd accumulation in *Arabidopsis* sink organs [30]. Phytochelatin synthase produces glutathione-derived Cd-chelating peptides in the cytosol [31], whereas membrane-related genes may help stabilize receptors and transporters required for stress signaling and ion movement. Endomembrane-specific dimerization of ABCG16 and ABCG25 controls the transport of ABA and ABA-glucosyl ester during osmotic-stress responses [32]. Cd exposure induced ABC, oligopeptide, nitrate and vacuolar iron transporter genes in tomato roots [33]. Taken together, our results hypothesized that TR might restricts Cd accumulation through cell-wall reinforcement, buffers oxidative damage through antioxidant and osmotic adjustment, and reprograms signaling and transport networks to sustain growth under Cd stress. Future work should directly test whether TR forms Cd complexes in maize tissues and identify the specific transporters or aquaporins that connect TR-regulated ROS signaling with Cd tolerance.

## 4. Materials and Methods

### 4.1. Plant Materials and Growth Conditions

The maize inbred line B73 was used as the experimental material. Seeds of uniform size and fullness were selected, surface-sterilized with 0.6% sodium hypochlorite solution for 10 min, and rinsed five times with distilled water. The seeds were then evenly placed in germination trays (34 × 25 × 4 cm; 48 seeds per tray) lined with moist toilet paper, covered with vermiculite saturated with water. After 48 h, when most seeds had developed radicles, 1/2 Hoagland nutrient solution was added [34]. The solution was aerated for 10 min and replaced every 2 days. The growth conditions were as follows: temperature 28~32 °C, relative humidity 60%~75%, light intensity 600 μmol·m^−2^·s^−1^, and photoperiod of 18 h light/6 h dark.

When seedlings reached the two-leaf stage, stress and exogenous substance treatments were initiated. Four treatments were established: CK (normal nutrient solution), TR (23 mmol/L trehalose), Cd (200 mg/L CdCl_2_·2.5H_2_O), and Cd+TR (200 mg/L CdCl_2_·2.5H_2_O + 23 mmol/L trehalose). The stated Cd treatment concentration refers to the hydrated salt and corresponds to approximately 0.876 mM. Each treatment comprised one cultivation tray containing 48 germinated seedlings. Seedlings were randomly sampled from each tray for physiological assays. The selection of 200 mg L^−1^ CdCl_2_·2.5H_2_O was consistent with our preliminary laboratory screening [35]. Preliminary phenotypic screening compared 16 mM, 23 mM, and 30 mM trehalose in combination with Cd, with a CK tray photographed alongside each treatment (Supplementary Figure S3). Based on this preliminary visual assessment, 23 mM trehalose was selected for subsequent experiments. This short-term hydroponic treatment was used to examine seedling responses to Cd injury. After 5 days of treatment, whole maize seedlings were collected for determination of growth parameters (plant height, root length, shoot dry weight, and root dry weight) and various physiological and biochemical indices. After 3 days of treatment, shoot tissues from CK, TR, Cd, and Cd+TR were collected, immediately frozen in liquid nitrogen, and stored at −80 °C for transcriptome sequencing analysis.

### 4.2. Growth Parameter Determination

After 5 days of treatment, the phenotypic characteristics of maize seedlings in each treatment group were photographed and recorded. Plant height (from the base to the highest leaf tip) and primary root length were measured using a ruler. Seedlings were divided into shoots and roots parts. Samples were oven-dried at 105 °C for 30 min, then at 80 °C to constant weight. Shoot dry weight and root dry weight were measured using an electronic balance, and total dry weight was calculated.

### 4.3. Physiological and Biochemical Parameter Determination

Chlorophyll content in leaves was determined spectrophotometrically following Wellburn [36]. The maximal photochemical efficiency of PSII (Fv/Fm) of the third fully expanded leaf was measured using a Handy PEA plant efficiency analyzer (Hansatech Instruments Ltd., King’s Lynn, UK). The determination method of superoxide anion (O_2_^−^), hydrogen peroxide (H_2_O_2_), and malondialdehyde (MDA) content was described in our previous work [14]. Superoxide dismutase (SOD) activity was assayed using the nitroblue tetrazolium photoreduction method. Catalase (CAT) activity was measured by monitoring the decrease in absorbance at 240 nm due to H_2_O_2_ decomposition, following Cakmak et al. [37]. Peroxidase (POD) activity was determined using the guaiacol method. Ascorbic acid (AsA) and reduced glutathione (GSH) contents were measured using commercial assay kits (Grace Biotechnology Co., Ltd., Suzhou, China; catalog Nos. G0201W (AsA) and G0206W (GSH); assay extracts react with the respective chromogenic reagents, followed by absorbance measurement at 534 nm for AsA-dependent ferric-ion reduction and bathophenanthroline complex formation, and at 412 nm for the GSH–DTNB reaction). Proline content was determined using the ninhydrin colorimetric method. Soluble protein content was measured using the Coomassie Brilliant Blue G-250 method. Soluble sugar content was determined using the anthrone colorimetric method. Root samples were first soaked in 10 mmol/L EDTA-2Na solution for 10 min to remove surface-adsorbed Cd. Both root and shoot samples were washed with deionized water, dried, ground, and passed through a 40-mesh sieve. A 0.2 g aliquot of the powder was digested using HNO_3_-HClO_4_ (83:17, *v*/*v*). Cd concentrations in shoots and roots were determined using Agilent 240FS AA flame atomic absorption spectrophotometer (Agilent Technologies, Santa Clara, CA, USA). Root cell-wall pectin, hemicellulose, and cellulose were fractionated and determined colorimetrically following Peng et al. [38], with modifications. Total cell-wall polysaccharide content was calculated as the sum of these fractions. Trehalose content and trehalase activity in leaves were measured using commercial assay kits (Grace Biotechnology Co., Ltd., Suzhou, China; catalog Nos. G0552F (trehalose) and G0554W (trehalase); trehalose is quantified by anthrone–sulfuric acid colorimetry at 620 nm, whereas trehalase activity is quantified from glucose released by substrate hydrolysis using a glucose oxidase-coupled color reaction).

### 4.4. Transcriptome Sequencing and Bioinformatics Analysis

Total RNA was extracted from shoot tissues of CK, TR, Cd, and Cd+TR (three samples per treatment; twelve samples in total) using a plant RNA extraction kit. RNA quality was assessed using a BioDrop microvolume spectrophotometer (Biochrom Ltd., Cambridge, UK) and 2% agarose gel electrophoresis. Qualified RNA samples were sent to Shanghai Personal Biotechnology Co., Ltd. (Shanghai, China) for cDNA library construction and high-throughput sequencing on the Illumina platform. Adapter and primer sequences and low-quality reads were removed before alignment. Approximately 36.57–46.41 million clean reads per sample were aligned to the maize B73 reference genome (Zm-B73-REFERENCE-GRAMENE-4.0) using HISAT2 (version 2.2.1) with default parameters [39]. Alignment analysis revealed that over 90% of the transcriptome sequencing data could be mapped to the genome, with the percentage of uniquely mapped reads exceeding 81% for all samples.

The resulting alignment files were converted, sorted, and indexed using SAMtools (version 1.17) [40]. Gene-level read counts were quantified using featureCounts (version 2.0.1) based on the corresponding genome annotation file. Differential expression analysis between groups was performed using the DESeq2 (version 1.40.2) package in R. Differentially expressed genes (DEGs) were identified using the criteria of at least a twofold change in either direction (FC ≥ 2 or FC ≤ 0.5; equivalent to |log_2_FC| ≥ 1) and false discovery rate (FDR) ≤ 0.05. GO enrichment analysis was performed on DEGs between the Cd and Cd+TR treatments, using the maize genome annotation as the background, with significant enrichment defined as Bonferroni-corrected *p* ≤ 0.05.

### 4.5. Data Processing

For each treatment, physiological measurements were performed in triplicate on material randomly sampled from three cultivation tray. Data are presented as mean ± standard error (SE). One-way analysis of variance (ANOVA), with treatment as the single factor, was conducted using SPSS 20.0 software. Descriptive statistics and tests of homogeneity of variance were obtained. Treatment means were compared using Duncan’s multiple range test at *p* < 0.05. Graphs were generated using Microsoft Excel 2010.

## 5. Conclusions

This study demonstrates that exogenous trehalose alleviates Cd toxicity in maize seedlings through coordinated physiological responses and transcriptomic adjustments. Under Cd stress, exogenous trehalose increased shoot and root dry weight and restored chlorophyll content. It reduced Cd concentrations in roots and shoots, accompanied by an increase in root cell-wall cellulose, while alleviating oxidative damage through reduced $O_{2}^{-}$, H_2_O_2_, and MDA contents and enhanced SOD and CAT activities. Transcriptome analysis further showed enrichment of cell communication, signal transduction, amino acid transmembrane transport and membrane-component categories, with expression patterns of *ZmTPS*/*ZmTPP* and antioxidant-related genes consistent with the physiological changes. Therefore, these findings concluded that trehalose supply could ameliorate the Cd-induced deleterious effects on maize seedling growth.

## Data Availability

The RNA-seq data have been deposited at NCBI (SRA; http://www.ncbi.nlm.nih.gov/sra (accessed on 9 October 2026)) under accession numbers PRJNA1531973.

## Supplementary Materials

The following supporting information can be downloaded at: https://www.mdpi.com/article/10.3390/plants15203117/s1, Figure S1: Effects of exogenous trehalose on Cd transfer coefficient in maize seedlings under Cd stress; Figure S2: Relative contents of polysaccharide components in maize root cell walls under different treatments; Figure S3: Preliminary phenotypic screening of trehalose concentrations in maize seedlings under Cd stress; Figure S4: Effects of exogenous trehalose on the expression of *TPP* genes in maize seedlings under Cd stress; Table S1: The summary of RNA-seq; Table S2: Primer sequences used for quantitative real-time PCR (qRT-PCR) analysis of *ZmTPP8* and *ZmTPP23*; Table S3: Differentially expressed genes identified in the treatment comparisons.
