# Supplementary material for: Physiological Responses and Transcriptomic Characteristics of Exogenous Trehalose Alleviating Cadmium Toxicity in Maize Seedlings
Source: Plants (Basel). 2026 Oct 12;15(20):3117. doi: 10.3390/plants15203117 (PMC13708718; doi:10.3390/plants15203117)
Supplement: Supplementary file 1 [file plants-15-03117-s001.zip › supplementary file.pdf]

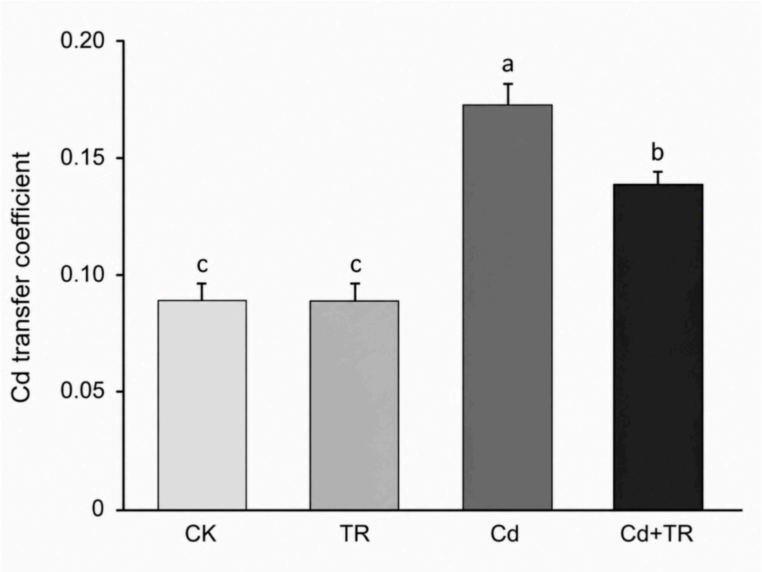

Figure S1. Effects of exogenous trehalose on Cd transfer coefficient in maize seedlings under Cd stress. CK, control; TR, 23 mM trehalose; Cd, 200 mg L^{-1} $CdCl_2 \cdot 2.5H_2O$; Cd+TR, 200 mg L^{-1} $CdCl_2 \cdot 2.5H_2O$ supplemented with 23 mM trehalose. Values are means ± SE (n = 3). Different lowercase letters indicate significant differences according to Duncan's multiple range test ($P < 0.05$).

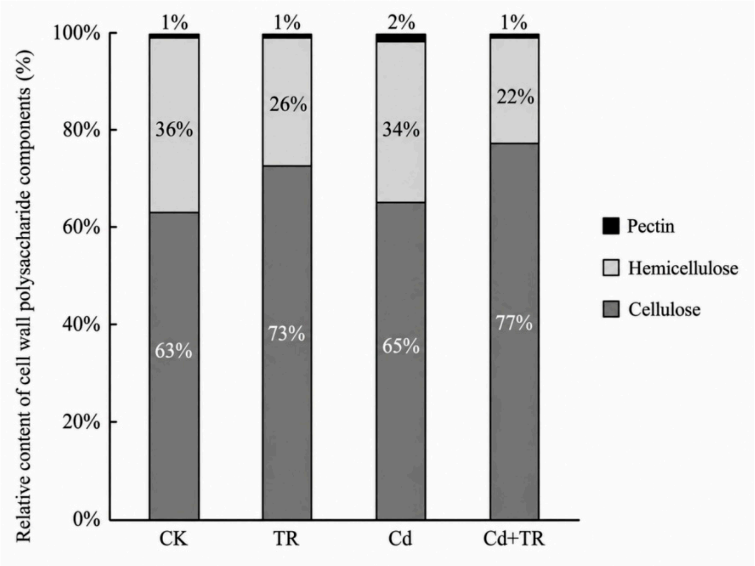

Figure S2. Relative contents of polysaccharide components in maize root cell walls under different treatments. CK, control; TR, 23 mM trehalose; Cd, 200 mg L^{-1} $CdCl_2 \cdot 2.5H_2O$; Cd+TR, 200 mg L^{-1} $CdCl_2 \cdot 2.5H_2O$ supplemented with 23 mM trehalose. Values are means ± SE (n = 3). Different lowercase letters indicate significant differences according to Duncan's multiple range test ($P < 0.05$).

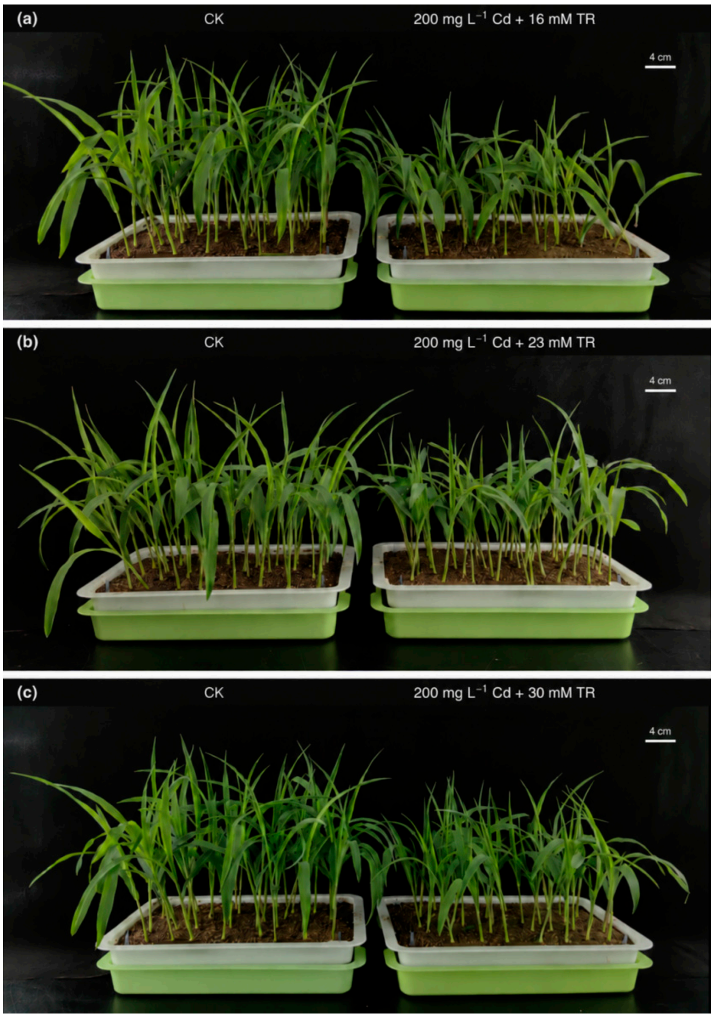

Figure S3. Preliminary phenotypic screening of trehalose concentrations in maize seedlings under Cd stress. Each panel shows CK (control) on the left and seedlings treated with 200 mg L^{-1} $CdCl_2 \cdot 2.5H_2O$ supplemented with (a) 16, (b) 23, or (c) 30 mM trehalose on the right. Scale bars = 4 cm.

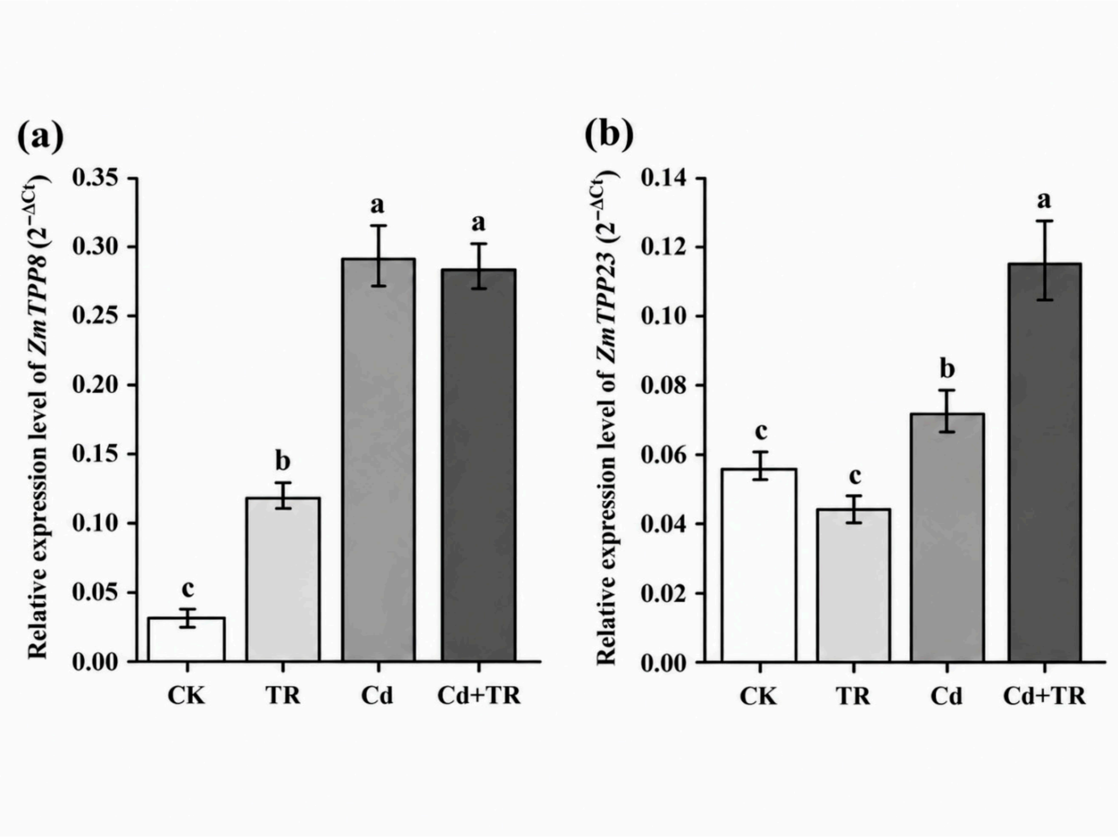

Figure S4. Effects of exogenous trehalose on the expression of *TPP* genes in maize seedlings under Cd stress. Relative expression levels of (a) *ZmTPP8* and (b) *ZmTPP23* were determined by qRT-PCR using cDNA templates obtained after 72 h of treatment. qRT-PCR amplification was carried out on a Bio-Rad real-time PCR system (USA) with TransStart® Tip Green qPCR SuperMix (TransGen Biotech, China; Cat. No. AQ141). *ZmACTIN* was used as the internal reference gene. CK, control; TR, 23 mM trehalose; Cd, 200 mg L^{-1} $CdCl_2 \cdot 2.5H_2O$; Cd+TR, 200 mg L^{-1} $CdCl_2 \cdot 2.5H_2O$ supplemented with 23 mM trehalose. Values are means ± SE (n = 3). Different lowercase letters indicate significant differences according to Duncan's multiple range test ($P < 0.05$). *ZmACTIN* was selected as the internal reference gene.

Table S1. The summol L-1ary of RNA-seq.

Sample name	Total reads	Mapped N/A	Unique mapping reads	Unique mapping%
CK_1	40372784	90.16%	33061430	81.89038933
CK_2	45922408	95.55%	37805306	82.32431104
CK_3	46408172	95.70%	37716630	81.27152692
TR_1	42042468	95.79%	35273090	83.89871403
TR_2	44081648	95.77%	37255798	84.51543826
TR_3	42113068	95.80%	35220688	83.63363125
Cd_1	36573776	95.85%	30653082	83.81164143
Cd_2	44888376	95.20%	37696050	83.97730851
Cd_3	38965662	95.46%	32569372	83.58480346
Cd+TR_1	36820240	95.68%	31234684	84.83020208
Cd+TR_2	39005954	95.64%	33187536	85.08325678
Cd+TR_3	39808704	95.51%	34200778	85.91281444

Table S2. Primer sequences used for quantitative real-time PCR (qRT-PCR) analysis of *ZmTPP8* and *ZmTPP23*.

	Gene ID	Primer	Sequence
ZmTPP8	Zm00001d029371	qTPP8-F	CATGACGGACGAGATGAGGG
		qTPP8-R	CCTCCGCCTTGCCGTT
ZmTPP23	Zm00001d052060	qTPP23-F	GAACCCCCAGGGCATCAG
		qTPP23-R	CTCACGCCGCCGCTATT
ZmACTIN		qACTIN-F	ATGTTTCCTGGGATTGCCGAT
		qACTIN-R	CCAGTTTCGTCATACTCTCCCTTG
